# Engagement in Self-measured Blood Pressure Monitoring Among Medically Underresourced Participants (the Reach Out Trial): Digital Framework Qualitative Study

**DOI:** 10.2196/38900

**Published:** 2023-04-07

**Authors:** Abby Katherine Hellem, Candace Whitfield, Amanda Casetti, Maria Cielito Robles, Mackenzie Dinh, William Meurer, Lesli Skolarus

**Affiliations:** 1 Department of Neurology Michigan Medicine University of Michigan Ann Arbor, MI United States; 2 Department of Emergency Medicine Michigan Medicine University of Michigan Ann Arbor, MI United States; 3 Davee Department of Neurology Feinberg School of Medicine Northwestern University Chicago, IL United States

**Keywords:** mobile health, mHealth, cardiovascular disease, hypertension, blood pressure, semistructured interviews, intervention engagement, social determinants of health, DBCI framework

## Abstract

**Background:**

Mobile health (mHealth) interventions serve as a scalable opportunity to engage people with hypertension in self-measured blood pressure (SMBP) monitoring, an evidence-based approach to lowering blood pressure (BP) and improving BP control. Reach Out is an SMS text messaging–based SMBP mHealth trial that aims to reduce BP among hypertensive patients recruited from the emergency department of a safety net hospital in a low-income, predominately Black city.

**Objective:**

As the benefits of Reach Out are predicated on participants’ engagement with the intervention, we sought to understand participants’ determinants of engagement via prompted SMBP with personalized feedback (SMBP+feedback).

**Methods:**

We conducted semistructured telephone interviews based on the digital behavior change interventions framework. Participants were purposively sampled from 3 engagement categories: high engagers (≥80% response to SMBP prompts), low engagers (≤20% response to BP prompts), and early enders (participants who withdrew from the trial).

**Results:**

We conducted interviews with 13 participants, of whom 7 (54%) were Black, with a mean age of 53.6 (SD 13.25) years. Early enders were less likely to be diagnosed with hypertension prior to Reach Out, less likely to have a primary care provider, and less likely to be taking antihypertensive medications than their counterparts. Overall, participants liked the SMS text messaging design of the intervention, including the SMBP+feedback. Several participants across all levels of engagement expressed interest in and identified the benefit of enrolling in the intervention with a partner of their choice. High engagers expressed the greatest understanding of the intervention, the least number of health-related social needs, and the greatest social support to engage in SMBP. Low engagers and early enders shared a mixed understanding of the intervention and less social support compared to high engagers. Participation decreased as social needs increased, with early enders sharing the greatest amount of resource insecurity apart from a notable exception of a high engager with high health-related social needs.

**Conclusions:**

Prompted SMBP+feedback was perceived favorably by all participants. To enhance SMBP engagement, future studies could consider greater support in the initiation of SMBP, evaluating and addressing participants’ unmet health-related social needs, as well as strategies to cultivate social norms.

## Introduction

Hypertension, the most important modifiable cardiovascular risk factor, affects nearly half of the adult population in the United States [1-5]. Many Americans have uncontrolled blood pressure (BP). In 2018, about 116.4 million (46%) adults had hypertension [6-8]. Black Americans have the highest prevalence of hypertension of any racial or ethnic group in the United States and are less likely to have their BP controlled than White Americans [8]. Hypertension disparities are also evident among low-income Americans, who have a higher prevalence, less awareness, and less treatment of BP and poorer BP control than other Americans [9]. Self-measured blood pressure (SMBP) monitoring is the regular measurement of BP by an individual outside of the clinical setting [10]. SMBP is effective in lowering BP and improving BP control, particularly when combined with other strategies, including behavioral counseling, education, and training [11-13].

Given the high penetrance of mobile phones, estimated at nearly 97% of Americans, mobile health (mHealth) interventions serve as a scalable opportunity to engage people with hypertension [14]. mHealth strategies can include reminders to measure BP, BP feedback, visualization tools, and telemonitoring. Specifically, telemonitoring allows people to obtain their BP readings, and these BP readings can also be transmitted to patients’ care teams, which has been shown to be more effective than SMBP alone [13,15].

The benefits of SMBP are predicated on engagement. It has been suggested that a standardized definition of engagement with digital behavior change interventions (DBCIs) may be based on the extent of usage and the subjective experience of the participant [16]. Within the confines of this qualitative study, we defined engagement as the frequency with which participants responded to prompts to self-report their BP via SMS text message. Using this initial objective measure of engagement (ie, SMBP), we then explored the more subjective experiences and measures of engagement. Despite many people having a home BP cuff, engagement in SMBP may be low [17-19]. Researchers have explored age, family history of hypertension, use of antihypertension medication, BMI, and smoking as factors associated with SMBP [20]. In this context, we sought to understand the determinants of engagement with SMBP based on the DBCI conceptual framework among patients with hypertension. These patients were recruited from the emergency department (ED) of a safety net hospital located in a low-income, predominantly Black city and were enrolled in Reach Out, a 1-year mHealth clinical trial to lower BP [16].

## Methods

### Overview

We conducted theory-based, semistructured interviews with participants in the Reach Out trial to understand their engagement with SMBP. The interviews were conducted following the conclusion of the Reach Out trial from June to September 2021. Interviews were between 30 and 60 minutes long.

### Reach Out Trial and SMBP

Reach Out was an SMS text messaging–based factorial clinical trial assessing behavioral interventions to reduce BP among the safety net ED patient population [21]. Participants of the Reach Out trial were required to have texting capability and were informed during the consent process that standard SMS text messaging rates could apply, depending on their cellular plan. Participants were randomized into 1 of 8 component arms consisting of varying intensity levels: (1) healthy behavior SMS text messaging (daily vs none), (2) SMBP monitoring (daily vs weekly), and (3) facilitated primary care provider appointment scheduling and transportation (yes vs no). Regarding SMBP monitoring, during ED enrollment, a BP cuff was distributed, and participants underwent training on BP cuff use and how to format their BP text message responses to be recognized by the SMS text messaging system as a BP reading (eg, 140/90). They were also given written materials about how to properly send text messages, take their BP, and the significance of hypertension. Participants were able to tailor the time of their text messages based on their preferences (ie, morning, afternoon, or evening). Participants were prompted to take their BP and text responses of their BP at daily or weekly intervals, depending on their randomized group assignment. If participants randomized to weekly intervals did not respond with an SMBP reading, they receive up to 2 additional reminders during the week. Each week, participants received a tailored feedback message based on their recent self-reported BP compared to normal BP thresholds (130/80 mmHg). Herein, the combination of these mechanisms will be referred to as SMBP+feedback. Additionally, participants may prompt the SMS text messaging system to provide a graph or list of their self-reported BP readings since their randomization. Graphs were sent through multimedia messaging services (a text message containing audiovisual material) only requiring the availability of a cellular network.

### Participants

We contacted 31 Reach Out trial participants randomized to the weekly or daily SMBP prompts, of which 13 (42%) participated in the semistructured telephone interviews. We performed purposeful sampling with an emphasis on variation in engagement to understand the determinants of engagement across trial participants [22]. Thus, we enrolled participants from 3 separate engagement categories: high engagers, low engagers, and early enders. High engagers were defined as participants who responded to 80% or more (n= 292+ responses for daily prompt recipients, n=42+ responses for weekly prompt recipients) of the SMBP prompts, while low engagers were defined as participants who responded to 20% or less (n= 73- responses for daily prompt recipients, n=10- responses for weekly prompt respondents) of the SMBP text prompts. Early enders were participants who formally withdrew themselves from the study, either by texting “STOP” or by directly contacting the research team, before their 12-month end date.

### Interview Guide

**Figure 1:**
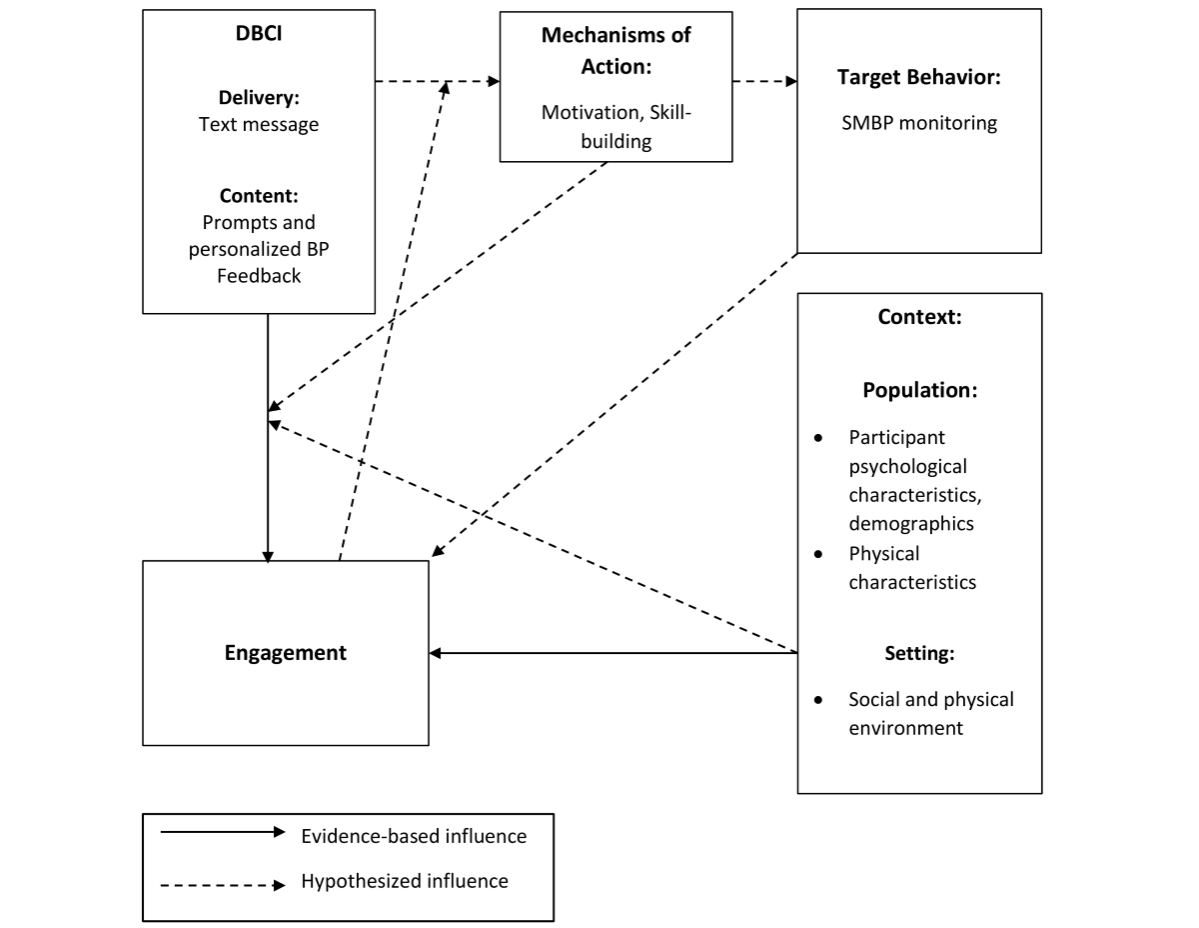
Simplified and tailored digital behavior change intervention (DBCI) framework. BP: blood pressure; SMBP: self-measured blood pressure.

We developed an interview guide based on a conceptual framework of engagement with DBCIs that was created from a synthesis of 117 articles (Multimedia Appendix 1). The framework proposes that engagement is directly influenced by the DBCI itself, including its content and delivery, as well as contextual factors pertaining to the user [16]. A simplified and tailored model of the DBCI framework is presented in Figure 1. For the development of our interview guide, we focused on the delivery and content of the intervention; the user’s physical and social environment; and demographic, physical, and psychological factors. We specifically queried: (1) intervention mode of delivery and complexity; (2) health-related social needs and their digital/general literacy levels; (3) users’ perception and experience engaging in SMBP; (4) users’ confidence to engage in SMBP; (5) the effects of community and familial support; and (6) access to quality cell-phone service, time/responsibilities, and digital redlining.

### Data Collection

Participants were given the option to complete the interviews either through web-based Zoom meetings approved by HIPAA (Health Insurance Portability and Accountability Act) or by telephone. All interview participants chose to complete the interview via telephone, which was conducted by a primary interviewer (author CW). A second study team member (author MCR) served as a real-time data collector who utilized a structured data collection form to conduct real-time transcription and note verbal clues such as sarcasm during the interviews [23]. All interviews were audio recorded and transcribed verbatim for analysis.

### Ethics Approval

This study was approved by the University of Michigan Institutional Review Board (HUM00138470). The Reach Out Trial was registered on ClinicalTrials.gov (NCT03422718). Informed consent was obtained from all participants.

### Data Analysis

We developed a codebook based on the DBCI engagement framework and conducted thematic analysis using ATLAS.ti qualitative data analysis software (ATLAS.ti Scientific Software Development GmbH). To establish intercoder agreement, 2 authors (AKH and AC) double-coded 4 (30%) of the transcripts and compared their results. Discrepancies in coding were discussed by the coders until an agreement was reached. With a shared understanding of the coding schema, the remainder of the transcripts were coded. The intercoder agreement was .98 Krippendorff c-alpha binary.

## Results

### Participant Characteristics

We conducted interviews with 13 participants, almost equally divided between White and Black participants. Characteristics of individuals who participated in the interviews, declined, or were unable to be reached, along with those of the Reach Out trial population, are shown in Table 1.

Given the nature of the semistructured interviews, not all questions may have been asked or answered by all participants; thus, the denominators in the results section may fluctuate.

**Table 1:** Participant characteristics^a^.

Characteristics		Interviewed participants (n=13)				Declined (n=18)		Reach Out study (n=488)
		High engagers (n=4)	Low engagers (n=4)	Early enders (n=5)				
Age (years), mean (SD)		65.8 (9.3)	50.25 (12.1)	46.6 (9.7)	44.2 (9.6)		45.5 (12.4)	
**Race, n (%)**								
	Black/African American	1 (25)	3 (75)	3 (60)	8 (44)		262 (54)	
	White/European American	3 (75)	1 (25)	2 (40)	8 (44)		201 (41)	
**Gender, n (%)**								
	Female	3 (75)	2 (50)	4 (80)	12 (67)		299 (61)	
	Male	1 (25)	2 (50)	1 (20)	6 (33)		189 (39)	
**Education, n (%)**								
	High school/GED^b^/ or less	0 (0)	3 (75)	2 (40)	10 (55)		227 (46)	
	Some college/trade school	2 (50)	1 (25)	2 (40)	3 (17)		169 (35)	
	University/advanced degree	2 (50)	0 (0)	1 (20)	5 (28)		92 (19)	
**Health status, n (%)**								
	Diagnosed with hypertension prior to Reach Out	4 (100)	3 (75)	4 (80)	14 (78)		385 (79)	
	Routine PCP^c^	4 (100)	3 (75)	5 (100)	13 (72)		380 (78)	
	Antihypertension medication (baseline)	4 (100)	2 (50)	4 (80)	7 (39)		273 (56)	

^a^Totals not equaling to total n indicate data that were missing or not applicable.

^b^GED: General Educational Development (a high school equivalency credential).

^c^PCP: primary care provider.

### Target Behavior

Most high engagers (3/4, 75%) and a couple of low engagers (2/4, 50%) had tried SMBP monitoring prior to participating in Reach Out either at home or at a pharmacy. On the other hand, most early enders (4/5, 80%) shared that they had not tried self-monitoring before, stating that they never thought they needed to, did not have a BP cuff, or forgot.

### DBCI Delivery and Content

#### Understanding of Intervention

All high engagers (3/3, 100%) stated that they understood how the intervention worked, whereas only a couple of low engagers (2/4, 50%) and early enders (2/5, 40%) reported understanding the intervention, both in terms of how to self-measure their BP and the frequency with which they were supposed to report their BPs. As 1 low engager shared:

No [I didn’t understand], I just did it when...whenever I got texted to do it (giggle). I mean, I understood when I was texted, but I couldn’t tell you if it was like once a month or, you know…

#### Mode of Delivery

Mode of delivery refers to the method in which the intervention was administered to the participants; in the Reach Out trial, it was via SMS text messaging. All participants (12/12, 100%) expressed being comfortable with text messaging and using a mobile phone in general.

#### Feedback

All high engagers (4/4, 100%) and early enders (5/5, 100%) and most low engagers (3/4, 75%) expressed liking the DBCI’s feedback messages that their BP reading had been received by the research team, sharing that these messages gave them a sense of confirmation and social support:

Well, I mean it-I think it was good. You know that was good for reassurance that I made…that I sent it in..because every once in a while, I’d have to go back and check, Oh, did I send my blood pressure in?

High engager

I felt like I had somebody with me that wasn’t present that was still on my team.

Early ender

Similarly, all high engagers (4/4, 100%) and early enders early enders (5/5, 100%) and most low engagers (2/3 67%) expressed liking feedback messages that interpreted their BP reading, letting them know if the BP they texted in was high, normal, or low. They said that these messages allowed them to reflect, provided them with a call to action to manage their BP, gave them a sense of reassurance, and generally equipped them with knowledge about their condition:

Um… [the messages were] great. Um...that way I knew I had to work a little bit better at either lowering or you know everything was okay.

High engager

Um, I appreciated those [messages]. I did. And then I was like, ‘What? Did I take my medicine or if I was eating too much salt, you know, it just kind of made me take a look at what I was doing.

Early ender

…That was helpful because, you know, I may’ve been eatin’ something I ain’t got no business [eating] (laughter).

Low engager

#### Control Features

Control features are defined as aspects of the DBCI that participants can adjust, and in doing so, provide a sense of control over their engagement. Only 1 (20%) early ender stated that they would have wanted more input into the types of messages they received. Similarly, another early ender shared that they would have wanted more input regarding the frequency of messages they received, stating they would have liked more messages.

#### Social Support Features

Social support features are aspects of the DBCI that provide assistance via interpersonal interaction. Participants were asked if they would have liked to participate in Reach Out with a partner. Among them, 2 (50%) high engagers shared that they already felt as though they had a partner participating with them because their spouse would encourage them to take their BP or take their BP with them. As 1 high engager stated:

Well, my…my wife stays on top of me when I do it, so I mean, I guess I already got [a partner], and I don’t complain.

Other participants shared diverse opinions on having a partner participate with them. Most early enders (3/5, 60%), a couple of low engagers (2/4, 50%), and 1 (25%) high engager shared that they would have liked a partner or noted the value of a partner. As the high engager put it, a partner would have provided them with additional support and would have added a sense of fun and competition to the intervention:

Um...it would’ve been great (laughing) to have someone doing it while I was doing it. […] Yeah. More support and … um…uh...yeah support and, you know making a game out of it, making it where it was challenging.

Participants who shared not wanting a partner cited that a partner would be unnecessary, unhelpful, or would not have made a difference in their participation. One high engager said:

It wasn’t necessary to have anybody doing it with me. I am doing it for myself.

An early ender declared:

Nah, it wouldn’t have mattered. I’d have done [Reach Out] anyways.

#### Professional Support Features

Professional support features are aspects of the DBCI that provide assistance via professional interaction. A couple of high engagers (2/4, 50%) and most early enders (3/5, 60%) expressed wanting more interaction with the research team. Early enders specified that they would have liked more in-person instruction on how to use the BP cuff and other BP-related resources:

I went through a lot of stress putting the pressure…you know learning how to use it, even with the instructions…so, I feel about if I came in and then they had shown me personally. Like I’m more of a visual type of guy…so, you would put it on for me and shown me how to put it on, then I’m good right there other than me turning it this way, or thinking it goes this way and it was uncomfortable this way. You know what I’m sayin’?

Early ender

Most (3/4, 75%) low engagers, however, stated that they would not have wanted more interaction with the research team. As 1 low engager put it, they would not have wanted more interaction because managing their BP is a behavior they engage in independently:

Um…I think [the amount of interaction I had with the research team] was fine because I pretty much maintain, you know, my blood pressure and all that kind of stuff. I can pretty much maintain myself, so, you know, but I like when y’all…when I talked to you guys. I enjoy it. I enjoy talking to you guys, you know, but…I don’t need nobody to come out and check on me and all that.

### Participant Context

#### Physical Context

Physical context refers to participants’ basic and technological resources, and the term cellular resources refers to participants’ capability to access and use mobile cellular technology. Nearly all participants (11/12, 92%) reported owning a smartphone, either an iPhone or Android, except for 1 (8%) participant, an early ender who owned a flip phone. All low engagers (3/3, 100%) and most high engagers (3/4, 75%) shared that it would be easy to obtain a new cell phone if theirs were to break, saying that they have insurance for their phones and easy access to a store. As 1 high engager said:

Well [replacing my phone would be] fairly easy. I mean, we’ve got the insurance on the phone, and there’s offices all over town here, so it wouldn’t be that hard.

However, another high engager stated that getting a cell phone would be a challenge due to financial constraints:

Um... [it would be] difficult (laughing). Um, because I’m considered low income and that’s a bill that I could…I probably wouldn’t have a cell phone on. My…I’m strapped most every month.

Most early enders (3/5, 60%) said that it would be challenging to obtain a new cell phone due to cost. As 1 early ender said:

[It’s] not very easy [to get a new phone]. You got to have money.

All high engagers (4/4, 100%) and early enders (4/4, 100%) and most low engagers (2/3, 67%) shared that there are many cell phone providers in their area. None of the high nor low engagers reported struggling to keep their cell phone service active. A couple of early enders (2/5, 40%) mentioned struggling to keep their service active, with one early ender citing it was because they forget to pay their bill. None of the participants from any engagement group reported changing their cell phone provider often.

#### Physical Resources

Physical resources are basic resources (ie, housing, transportation, and food) that significantly influence individuals’ quality of life and health outcomes. All participants (13/13, 100%) shared that they have stable housing.

#### Transportation

Most high engagers (3/4, 75%) and all early enders (5/5, 100%) shared that they have access to regular transportation; however, 1 (25%) high engager shared that their transportation had been “shaky.” Low engagers reported mixed access to transportation, with a couple (2/4, 50%) saying that they sometimes have trouble accessing transportation. As a low engager put it:

Yes, transportation is a problem sometimes, yes ’cause I don’t…I don’t drive. I don’t know how to drive (laughter). I have to rely on my kids, my neighbor, somebody.

#### Food

A couple of high engagers (2/4, 50%) and most of the early enders (3/5, 60%) shared that they worried that their food would run out before they got money to buy more. In contrast, most (3/4, 75%) low engagers reported not worrying that their food would run out. Most high (3/4, 75%) and low (3/4, 75%) engagers shared that they never actually ran out of food. On the other hand, most early enders (3/5, 60%) did experience running out of food.

#### Utilities

None of the high engagers expressed receiving notice that their utilities would be turned off. In contrast, 1 (25%) low engager and a couple of early enders (2/5, 40%) shared they had received notice that their utilities would be turned off. Two participants, 1 (25%) low engager and 1 (20%) early ender, went on to clarify that they were able to avoid the services being shut off.

#### Social Context

Social context is defined as the participants’ cultural and social normative environment in relationship to SMBP.

##### Perception of Others Wanting Them to Engage in SMBP Monitoring

All participants (12/12, 100%) shared that they believe that the people who care about them want them to monitor their BP. Participants shared that they believed others wanted them to self-monitor their BP for a variety of reasons, including their current health status and behaviors (“They’re worried about how high my blood pressure is and I’m [...] not taking medications for it right now,”) a near-death experience (“They almost lost me...uh, a year ago), wanting to make sure they will stay healthy in the future (“Uh, yeah, yeah…’Cause they don’t want nothing to happen to [me]. They don’t want [me] to die), and their relationship with the people who care about them (“Because they’re my children and I’m their mother.”)

##### SMBP Monitoring Social Normative Environment

Most high engagers (3/4, 75%) shared that they know others who also engage in SMBP monitoring; however, most low engagers (3/4, 75%) and early enders (3/5, 60%) did not know anyone engaging in SMBP monitoring. All high engagers (4/4, 100%) and the majority of low engagers (3/4, 75%) and early enders (4/5, 80%) stated that their friends and family knew about their participation in Reach Out and were supportive of their participation:

Oh, yes, they did [know about my participation]…They thought it was good and they also...um, each time that you all sent a graph…they wanted a copy, so we had a thread going and so they got a chance to see the up and down as well.

High engager

Uh…everyone was kinda like glad that I was doing something about [my blood pressure]. Glad that somebody was showing me how to wear my cuff, reminding me to take my press…blood pressure medicine, and-and let me see that it could be higher or lower. You know what I’m saying? […] So, it was a lot of people that was…that was kind happy that-that I was able to [participate].

Early ender

#### Psychological Context

Psychological context refers to the participants’ mental and emotional state in relationship to SMBP.

##### Perceived Importance of Self-monitoring

Overall, most high engagers (3/4, 75%) and all early enders (5/5, 100%) said that checking their BP was important to them:

...Checking my blood pressure is important to me ’cause…uh if it gets too high or it gets too low, I could be in trouble and…um blood pressure is the easiest way for black men to die these days from heart attacks, so it’s important that you keep up with your blood pressure.

Early ender

[Checking blood pressure is important]…um just to make sure your blood pressure is, you know do-doing great. You know, you don’t want it to be too high because, you know…With me, sometimes mine elevates high…really high…And…uh, you know if mine’s too high and my medicine does not control it, I’m gonna go to the hospital.

High engager

In contrast, low engagers reported mixed perceived importance of taking their BP; half of the low engagers (2/4, 50%) shared that they found checking their BP was important, while the other half (2/4, 50%) expressed that monitoring their BP is less important to them. As 1 low engager put it, monitoring their BP was less important because their BP had improved:

Five [out of 10 important]…’cause I just feel like, you know, checking it when you...when you...when you need to check it, but if nothing’s wrong with you, why would you be checking it?

All participants (12/12, 100%) shared that their health is very important to them.

##### Experience of Self-monitoring

All high engagers (4/4, 100%) and early enders (3/4, 75%) and most (3/4, 75%) low engagers shared that SMBP monitoring makes them feel positive or neutral emotions, including good, secure, aware, and responsible:

It made me feel really more responsible…’Cause…uh, I don’t have really no kids, like I just picked up some bills that I have to be responsible for now, but it made me more responsible because…uh you know you could just take a pill and eat the wrong thing and your blood pressure goes up. You know what I’m saying? That’s-that’s harmful, so it made me make sure I take my pressure every day, sometimes three times a day. You know what I’m saying? It made me pay attention to it, and I had the cuff from-from y’all to even take it…[Early ender]

It makes me feel good that I’m actually, you know, taking control of it a little bit there and makin’ sure I’m doing what I’m supposed to be doing and I’m not gonna have a stinkin’ stroke, you know?

Early ender

A few participants across all levels of engagement noted that how they feel while self-monitoring is dependent on their BP reading. One high engager said:

Well, when it’s where it should be, it makes me feel pretty darn good, but...it kind of bothers me when it’s like it was today, okay?

##### Self-perceived Barriers

Most low engagers (3/4) did not self-identify barriers to their participation in Reach Out. One low engager identified not being home as a barrier:

Sometimes...Sometimes if I’m not home, I might wait, that’s...that’s the only thing that, you know.

In contrast, the majority of early enders (3/5, 60%) identified barriers to their participation, including work and comorbidities (“depending on what time of the day it was, if I was working or not”). Another early ender said:

Just physical…during the study a couple of times, I had a hand surgery and an elbow surgery…So it was kinda hard then.

A visual comparative summary of these results between engagement groups is shown in Table 2.

**Table 2:** Comparison of engagement groups.

Variables		High engager	Low engager	Early ender
**Target behavior**				
	Tried SMBP^a^ monitoring prior to Reach Out	+^b^	+/−^c^	−^d^
**Design and delivery**				
	Understanding of the intervention	+	+/**−**	−
	Mode of delivery	+	+	+
	Feedback messages	+	+	+
	Control features	+	+	+
	Interaction with the research team	+/**−**	+	−
	Social support (enroll with partner)	+	+/**−**	+
**Physical context**				
	Smartphone ownership	+	+	+
	Ease of obtaining a new cell phone	+	+	−
	Cell phone providers in the area	+	+	+
	Stable cell phone service	+	+	+
	Stable housing	+	+	+
	Transportation access	+	+/**−**	+
	Food security	+/**−**	+	−
	Utility services	+	+	+
**Social context**				
	Believe others want them to self-monitor their BP^e^	+	+	+
	Know others who self-monitor their BP	+	−	−
	Others know about their participation in Reach Out	+	+	+
**Psychological context**				
	Importance of overall health	+	+	+
	Importance of monitoring BP	+	+/**−**	+
	Experience self-monitoring BP	+	+	+
	Absence of perceived barriers to participation	+	+	−

^a^SMBP: self-measured blood pressure.

^b^+: majority satisfaction/agreement/have resource.

^c^+/−: split satisfaction/agreement/resources attainment.

^d^−: majority dissatisfied/disagree/absence of resource.

^e^BP: blood pressure.

## Discussion

### Principal Results

We conducted a qualitative study to understand engagement with a prompted SMBP+feedback mHealth intervention among people with hypertension who were recruited from a safety-net ED. Overall, participants perceived their overall health and SMBP as important, were satisfied with the SMBP+feedback design of the DBCI, and did not have barriers to SMS text messaging access. Our results suggest that addressing factors including the capacity for personalization, enhanced SMBP monitoring enrollment procedures, and additional social and health-related social needs support may increase SMBP engagement.

Challenges in digital health literacy and mHealth are particularly prevalent among demographic groups adversely impacted by disparities in cardiovascular care [24]. These inequities can be further exacerbated by digital redlining, which presents unique challenges ranging from the affordability of individual technologies to the absence of basic infrastructure in marginalized communities, particularly notable with mobile applications [25]. A strength of Reach Out is that it was SMS text messaging–based and did not require a smartphone.

Despite employing a community-based participatory research approach for the design of SMBP instructions and SMBP prompts and feedback [26], some low engagers and early enders did not understand the purpose, frequency, or how to use the BP cuff. High engagers, in comparison to low engagers and early enders, expressed the greatest understanding of how SMBP was intended to help lower their BP. High engagers also represented the group with the highest level of completed formal education and had participated in SMBP monitoring previously. Thus, the enrollment strategies we used to introduce SMBP may be best suited for individuals with high levels of formal education or those who engaged in SMBP in the past. These conclusions further support the need to examine elements of enrollment to make them more suitable for individuals of all education levels and SMBP experience. Further, optional longitudinal technical support may be needed to increase engagement.

Participants’ unmet health-related social needs emerged as a theme associated with SMBP engagement. Early enders experienced more health-related social needs, including food insecurity and financial resources, as demonstrated by the ease of obtaining a new cell phone, than their counterparts. Unmet social needs may serve as additional barriers to SMBP monitoring by creating stress, introducing competing priorities, and reducing leisure time [27]. However, 1 (8%) of the participants had many unmet health-related social needs but was highly engaged in SMBP. Thus, unmet health-related social needs do not preclude engagement with SMBP. Our findings from the early enders suggest that additional resources to address unmet health-related social needs, such as information on community resources or community health worker support [28-31], may be needed for some participants.

Social norms may be another factor that influences participation. High engagers differed from low engagers and early enders in that most high engagers knew someone who engages in SMBP monitoring. Knowing others who engage in self-monitoring may aid in creating social norms that encourage participants to check their own BP. Future strategies to encourage SMBP could include support within participants’ social networks or support from others engaging in SMBP.

### Limitations

Our study has several limitations. First, women were overrepresented across all engagement levels. Men experience unique social norms that have reverberating impacts on other facets of life. The information gained from these interviews may not fully capture the barriers and facilitators that impact men’s engagement in SMBP. The study had a small sample, which limits some of the generalizability. With this small sample size of interviewed participants, this is a hypothesis-generating study. Further studies are needed to confirm the findings with a larger number of participants across each of the engagement groups. Consequently, we did not explore certain topics such as differences in engagement by smartphone type or digital literacy. However, within this small sample, different themes were identified between engagement groups. Finally, there is very little literature defining engagement categorization; thus, thresholds in determining engagement categorization were based on the best available literature [18] but ultimately not determined with statistical methods.

### Conclusions

Participants found this SMS text messaging–prompted SMBP+feedback mHealth intervention to be satisfactory. The tailored BP feedback was particularly appreciated. Participants who were high engagers knew others who engaged in SMBP, and overall, participants were open to engaging in SMBP with a partner. In fact, many had done so independently of Reach Out. The importance of hypertension literacy and the skills to measure BP are critical to ensuring engagement with SMBP. Finally, overall unmet health-related social needs increased as SMBP engagement decreased. Thus, prompted SMBP+feedback with the capacity for personalization, enhanced enrollment procedures, and additional social and health-related social needs support may further facilitate participant engagement in SMBP.

## Appendix

Multimedia Appendix 1 Participant interview guide.

## Abbreviations

BP: blood pressure
DBCI: digital behavior change intervention
ED: emergency department
HIPAA: Health Insurance Portability and Accountability Act
mHealth: mobile health
SMBP: self-measured blood pressure
SMBP+feedback: self-measured blood pressure with personalized feedback

## Data Availability

The data sets generated and/or analyzed during this study are available from the corresponding author upon request.
