# Supplementary material for: Engagement in Self-measured Blood Pressure Monitoring Among Medically Underresourced Participants (the Reach Out Trial): Digital Framework Qualitative Study
Source: JMIR Cardio. 2023 Apr 7;7:e38900. doi: 10.2196/38900 (PMC10131992; doi:10.2196/38900)
Supplement: Multimedia Appendix 1 [file cardio_v7i1e38900_app1.docx]

**Reach Out Engagement Interview Guide**

| **PART 1**  **Construct/Concept: Warmup / Introduction** |
| --- |
| **First, we would like to learn about how you use your phone.** |
| 1. Do you like texting? **DBCI-Delivery-Mode of Delivery** 2. How often do you text? **DBCI-Delivery-Mode of Delivery** 3. Were you comfortable with texting before joining the Reach Out Study? **DBCI-Delivery-Ease of use** 4. What kind of cell phone do you have? **Context-setting-physical**  - (a) Is this a smart phone? - (c) Can you download apps to your phone?  1. Do you use your phone to:    - Make calls    - Email    - Access the internet    - Apps    - Text    - Something else: 2. Did you understand how often you were supposed to take your blood pressure and text it back to the Reach Out team? **Mechanisms of action-knowledge**    - If not, how could we have explained this better? 3. Would you have wanted more say in the **type** of text messages you received from us? **DBCI-Delivery-Control features** 4. Would you have wanted more say in the **amount** of text messages you received from us? **DBCI-Delivery-Control features**    - If yes, what is your preferred amount of text messages?    - Were there some weeks you would have wanted more messages and some weeks fewer messages? |
| **PART 2**  **Construct/Concept: Self-monitoring blood pressure** |
| **Next we would like to learn more about how you feel about your health, and monitoring your own blood pressure.** |
| 1. On a scale of 1-10 how important is your overall health to you? **Context-population-psychological-personal relevance, experience of wellbeing** 2. How important is checking your blood pressure to you? **Context-population-psychological-personal relevance, experience of wellbeing**    - Why or why not? 3. Do you feel that the people who care about you want you to monitor your blood pressure?    - Why or why not? 4. Do you have friends or family members who monitor their own blood pressure? 5. Had you tried self-monitoring your blood pressure before joining the Reach Out Study? **Target behavior**    - If yes, how was that experience for you?    - If no, had you ever considered self-monitoring your blood pressure? 6. How helpful would it be to have friends or family participate in Reach Out with you? **DBCI-Content-social support features** 7. Did your friends and family know that you were a part of the Reach Out Study?    - If yes, how did they feel about your participation? 8. How does self-monitoring your blood pressure make you feel? **Engagement-affect**    - Why does self-monitoring your bp make you feel this way? 9. How does knowing your blood pressure numbers make you feel? **Engagement-affect** 10. Is there anything in your life that makes it hard for you to take your blood pressure and text it in? **Context-Population & setting**     - If yes, what may make this difficult? 11. After you texted in your blood pressure, you received a message saying: “Thank you for your response! REACH OUT has received your most recent BP.” How helpful did you find these responses? **DBCI-Delivery-Content** 12. After you texted in your blood pressure, you received a message saying: “One of your recent BP’s was XX/XX. Both the top and bottom numbers are either normal or high. Keep working to lower your BP with meds, eating healthy, and exercise.” How helpful did you find this response? **DBCI-Delivery-Content** |
| **Part 3**  **Construct/Concept: Barriers to participating** |
| **Now we would like to learn more about some of the other barriers that may have made it difficult for you to participate in reach out:**  **__________________________________________________________________________________**   1. Has your employment changed since you started Reach Out a year ago? 2. Are you allowed to use your phone while at work? **Context-setting-physical & social** 3. Have you ever found it difficult to keep your cell-phone service active? **Context-setting-physical**    - How has this affected you? 4. Do you change your cellphone or cellphone service often? **Context-setting-physical**    - What may influence you to make a change to your cellphone service or cellphone? 5. If your phone was to break today, how easy would it be to obtain a new device? **Context-setting-physical**    - If not easy, what barriers would you have to face to get a new device? 6. Are there many options for cell phone providers in your area? **Context-setting-physical**    - Who is your cell phone provider? 7. What is most important to you when choosing a new cellphone plan? 8. Did you show your blood pressure readings to your doctor?    - If yes, were any changes to your medicine made?    - If no, why? No doctor? Did not remember? 9. Did you show the graph of your blood pressure readings to your doctor?    - If yes, were any changed to your medicine made?    - If no, why? |
| **Part 4**  **Construct/Concept: Social determinants of health** |
| **We know that people’s lives are complicated and that sometimes parts of people’s lives make it harder for them to have a healthy lifestyle right now. We have a few questions about these things:** |
| 1. What is your housing situation today? **Context-setting-physical** 2. Within the past 12 months, have you been worried that your food would run out before you got money to buy more? **Context-setting-physical** 3. Within the past 12 months, has the food you bought run out before you got money to get more? **Context-setting-physical** 4. In the past 12 months, has lack of transportation kept you from medical appointments, meetings, work or from getting things needed for daily living? (Check all that apply) **Context-setting-physical** 5. In the past 12 months has the electric, gas, oil, or water company threatened to shut off services in your home? **Context-setting-physical** |
| **Part 5**  **Construct/Concept: Views on research team** |
| 1. Would you have preferred more interaction with the study team? **DBCI-Delivery-Professional support features/interactivity**   Probe: If yes, do you have an idea of how you would have wanted to interact with the study team more?   1. How important is it for you for you to have a research team that resembles people of your community? **DBCI-Delivery-Personalization** |
| **Demographics** |
| - Do you identify as: **Context-population-demographic**   - Man   - Woman   - Other   - Prefer not to say - How old are you? **Context-population-demographic** - How much school have you completed? **Context-population-demographic** - In general, would you say that your health is excellent, very good, fair, or poor? |
